# Development and validation of a national reference material system for quality control of chikungunya virus nucleic acid detection assays

**DOI:** 10.1128/jcm.01543-25

**Published:** 2026-05-06

**Authors:** Tingting Ma, Lan Zhao, Cong Luo, Yixi Wang, Yabin Tian, Sihong Xu

**Affiliations:** 1 Institute for In Vitro Diagnostics Control, National Institutes for Food and Drug Control 12540, Beijing, China; 2 State Key Laboratory of Drug Regulatory Science 12540, Beijing, China; Mayo Clinic Minnesota, Rochester, Minnesota, USA

**Keywords:** chikungunya virus, reference material, nucleic acid detection, standardization, digital PCR, quality control, molecular diagnostics

## Abstract

The absence of a World Health Organization (WHO) international standard for Chikungunya virus (CHIKV) nucleic acid testing has long impeded the comparability of diagnostic results across different assays and platforms. To address this critical gap in quality assurance, we developed and rigorously characterized a national reference material system consisting of a quantified standard and a validation panel for CHIKV molecular detection. These materials were developed using intact, inactivated CHIKV particles to closely mimic clinical extraction and amplification. The assigned value of the standard was determined through a robust multi-laboratory study using eight digital polymerase chain reaction (dPCR) platforms, yielding a traceable concentration of 7.60 ± 0.14 log_10_ copies/mL (*k* = 2). Extensive evaluation using nine kits demonstrated 100% concordance for both positive and negative samples. The materials exhibited excellent repeatability (coefficient of variation, CV <5%) and consistent detectability (≥95% positivity rate) at the claimed limit of detection across all kits. In summary, this study developed a well-characterized reference material system to support the standardization, development, and quality control of CHIKV nucleic acid assays. The availability of this reference material system addresses a longstanding limitation of results across different platforms and geographical regions. Their implementation is expected to facilitate assay optimization and improve the consistency of surveillance data, thereby strengthening preparedness and the accurate identification of cases, especially in non-endemic areas at risk of importation.

**IMPORTANCE:**

The absence of a World Health Organization (WHO) international standard for Chikungunya virus (CHIKV) nucleic acid testing has long compromised result comparability and global outbreak response. Here, we established a national reference material system derived from intact, inactivated CHIKV particles—authentically mirroring the entire clinical workflow. Covering both Asian and East/Central/South African (ECSA) genotypes, this system mitigates false-negative risks caused by viral genetic diversity. Assigned via multi-laboratory dPCR and validated across nine commercial kits, it demonstrated 100% concordance, ≥95% detectability at LoD, and coefficient of variation (CV) <5%. This traceable, genotype-inclusive, workflow-authentic benchmark enables, for the first time, unified performance alignment for regulatory review, manufacturer quality control, and inter-laboratory comparison. It fills a critical gap in domestic quality assurance while providing a technical template for future WHO standardization efforts. Widespread adoption will accelerate reliable diagnostics, strengthen surveillance comparability, and support timely clinical decisions in both endemic and importation-prone settings.

## INTRODUCTION

Chikungunya fever (CHIK) is an acute infectious disease caused by Chikungunya virus (CHIKV) primarily transmitted through the bite of infected *Aedes* mosquitoes. It is widely prevalent in tropical and subtropical regions globally, with a notable expansion of its geographic distribution in recent years (1, 2). CHIKV infection can lead to diverse clinical manifestations, such as fever, severe arthralgia, and rash, with symptoms often presenting in distinct disease phases. Nucleic acid testing and serological assays are currently the primary laboratory diagnostic methods (3, 4). Currently, the World Health Organization (WHO) has not yet established an international standard for CHIKV nucleic acid testing, which impedes the standardization and comparability of test results across different regions and platforms.

In China, nucleic acid testing was incorporated into the definitive diagnostic criteria for CHIK in the *Diagnostic Criteria for Chikungunya Fever* (WS/T 590-2018) issued in 2018 (5). The recently released *Chikungunya Fever Diagnosis and Treatment Protocol* (*2025 Edition*) further underscores its central role in early diagnosis and outbreak response (6). Owing to its early detection window, high sensitivity, and strong specificity, nucleic acid testing is established as an indispensable tool for diagnosing acute CHIKV infections (7, 8).

However, CHIKV nucleic acid testing still faces several challenges. Suboptimal sensitivity in certain commercial kits can lead to false-negative results, particularly during low-level viremia, increasing the risk of missed diagnoses and subsequent transmission (9). Furthermore, the high degree of genetic homology among alphaviruses not only causes serological cross-reactivity but also raises the possibility of false-positive results in nucleic acid testing due to non-specific amplification or cross-hybridization (10). Although emerging technologies, such as isothermal amplification and attenuated total reflection (ATR-FTIR) coupled with artificial intelligence, have shown promise for rapid detection, their performance lacks unified evaluation standards (11, 12). To address these issues, this study developed a national reference material system for CHIKV nucleic acid testing aiming to provide a scientifically robust measurement benchmark for the development, evaluation, and quality control of related assays.

## MATERIALS AND METHODS

### Material preparation and characterization

The national standard and the positive components of the national reference panel were prepared from heat-inactivated CHIKV strains. The negative samples (N1–N14 and N16) from the national reference panel were inactivated by heating (56°C, 30 min) or β-propiolactone (BPL) treatment (4°C, 48 h). The strains used include both the East/Central/South African (ECSA) genotype (for the standard and samples P1–P5) and the Asian genotype (for sample P6), which were preserved laboratory strains or clinically isolated strains obtained from collaboration partners.

To verify the strain identity and genotype, all reference materials were subjected to metagenomic next-generation sequencing (mNGS). Briefly, total nucleic acid was extracted, reverse-transcribed to cDNA, and amplified using random priming and sequence-independent single-primer amplification (SISPA) for library construction. Paired-end sequencing (2 × 150 bp) was performed on an Illumina NovaSeq 6000 platform; raw reads were processed for quality control, host sequence filtering, and taxonomic classification using Trimmomatic, Kraken2, and Bracken, respectively. This analysis confirmed the species and genotype of the raw materials for the reference material system.

To improve stability, the national standard was prepared by lyophilization. The inactivated virus suspension was formulated, dispensed, lyophilized under controlled conditions, and finally sealed under dry nitrogen. Detailed lyophilization parameters are provided in Table S1. The national standard consists of a single preparation intended for evaluating the repeatability and limit of detection (LoD) of CHIKV nucleic acid assays. The reference panel comprises 22 vials (0.5 mL each), including six positive samples derived from diverse sources with pre-set different concentration levels for assessing analytical sensitivity and 16 negative samples (for details, see Table 1) comprising (i) molecularly related alphaviruses (e.g., Sindbis virus), (ii) clinically similar pathogens (e.g., Dengue virus and Zika virus), and (iii) cross-reactivity controls (including other common pathogens and negative matrices) strictly selected following the latest domestic and international disease management and technical guidance principles (5, 6, 13–16) to systematically evaluate the specificity and reliability of detection kits in real-world, complex sample backgrounds.

**TABLE 1:** Source materials and specifications of the CHIKV national reference material system

Type	ID	Type/species of pathogen	Raw material concentration	mNGS sequencing results
Positive reference material	P1	CHIKV culture	10^10^ copies/mL	ECSA genotype
	P2	CHIKV culture	10^10^ copies/mL	ECSA genotype
	P3	CHIKV culture	14.9 (CT)^*a*^	ECSA genotype
	P4	CHIKV culture	14.9 (CT)	ECSA genotype
	P5	CHIKV culture	<20 (CT)	ECSA genotype
	P6	CHIKV culture	17.19 (CT)	Asian genotype
Negative reference material	N1	Zika virus culture	1.6 × 10^9^ copies/mL	Zika_virus
	N2	Dengue type one culture	9.61 × 10^10^ copies/mL	Dengue_virus_type_1
	N3	Dengue type two culture	6.13 × 10^10^ copies/mL	Dengue_virus_type_2
	N4	Dengue type three culture	1.18 × 10^10^ copies/mL	Dengue_virus_type_3
	N5	Dengue type four culture	5.61 × 10^10^ copies/mL	Dengue_virus_type_4
	N6	Tahyna virus culture	14.27 (CT)	Tahyna virus
	N7	Clinical samples of human parvovirus	20 (CT)	Human parvovirus
	N8	Epstein-Barr virus culture	18 (CT)	Epstein-Barr virus
	N9	Measles virus culture	1.45 × 10^8^ copies/mL	Measles virus genotype A
	N10	Clinical samples of cytomegalovirus	N/D^*b*^	Cytomegalovirus
	N11	Japanese encephalitis	8.61 × 10^9^ copies/mL	Japanese encephalitis
	N12	West Nile virus culture	3.83 × 10^9^ copies/mL	West Nile virus
	N13	Yellow Fever Virus Culture	6.1 × 10^7^ copies/mL	Unable to detect
	N14	Sindbis virus culture	2.55 × 10^7^ PFU/mL	Sindbis virus
	N15	Negative plasma	N/D	Negative
	N16	Clinical samples of herpes simplex virus	16 (CT）	Herpes simplex virus type 2
National standard	S	CHIKV culture	10^10^ copies/mL	ECSA genotype

^*a*^ Cycle threshold.

^*b*^ N/D, concentration of the raw material not determined.

### Homogeneity and stability of the national reference material system

The homogeneity, short-term stability, and freeze-thaw stability of the national standard were assessed via a laboratory-developed digital PCR method. Homogeneity was assessed with 10 randomly selected vials. Short-term stability was tested after storage at 4 and 25°C for 1–3 days. Freeze-thaw stability was examined using three independent vials subjected to one, two, and three cycles, respectively.

The homogeneity and stability of the reference panel were evaluated using two commercial (but non-approved) CHIKV nucleic acid detection kits (fluorescence-based PCR method; for details, see kits 6 and 8 in Table S3). Homogeneity was assessed with three sample sets; short-term and freeze-thaw stability were evaluated under identical conditions as those used for the national standard.

### Cooperative calibration and quality standard determination

Ten participating laboratories assigned values to the national standard using their optimized in-house dPCR quantification protocols under a unified operating procedure. A total of eight different dPCR platforms were employed (for details, see Table S2), including the QIAcuity One 5-Plex System (three labs), QX-200 System (three labs), and TD-1 System (two labs). Additional platforms were each used by one lab. Each laboratory’s reported concentration was derived from multiple independent replicate measurements (e.g., replicate wells).

### Collaborative characterization of the national reference material system

The national reference material system was evaluated through a multi-center study using nine commercial CHIKV nucleic acid detection kits (research-use-only; see Table S3 for the complete list). Performance characterization included positive agreement, negative agreement, repeatability, and LoD assessments.

Positive (P1–P6) and negative (N1–N16) reference materials were diluted to 1 mL using negative human serum/plasma or manufacturer-recommended diluent. Each sample underwent two independent extraction processes, with one test per extract. Repeatability was assessed by testing the national standard diluted to two concentrations: a medium- high level and a low concentration (1.5–4× the LoD) using negative human plasma or compatible diluent, with 10 technical replicates per concentration. The LoD was determined through serial dilution of the standard in negative plasma/diluent, with each dilution tested in triplicate. The highest dilution that yielded consistently positive results was identified as the preliminary LoD. The preliminary LoD was subsequently confirmed through 20 independent replicate tests at this concentration, with a required positivity rate of ≥95% (19/20 positive). The experimentally determined LoD was then compared to the manufacturers’ claimed LoD (as stated in their product insert) to verify if the kit met its claimed sensitivity.

### Statistical analysis

Statistical analysis was performed using Microsoft Excel 2016. The homogeneity and stability of the reference panel were evaluated by the CV, and a variation rate ≤5% was considered acceptable. The homogeneity of the national standard was analyzed by *F*-test. If the calculated *F*-value was lower than the critical *F*-value, the homogeneity of the reference standard was confirmed. The stability of the reference standard was evaluated by one-sample *t*-test. The certified value was calculated at a 95% confidence level, and the expanded uncertainty was calculated with a coverage factor of *k* = 1.96.

## RESULTS

### Construction and composition of the national reference material system

The national reference material system was successfully developed to address the lack of CHIKV nucleic acid testing benchmarks, with its final compositions detailed in Table 1. The panel consists of six positive samples (P1–P6) and 16 negative samples (N1–N16), while the national standard comprises a single lyophilized material derived from an ECSA genotype CHIKV strain. One positive panel member (P6) is derived from an Asian genotype strain to cover major circulating genetic variants.

### Evaluation of homogeneity and stability

The homogeneity and stability of the national reference material system were rigorously evaluated. The national standard assessed with a lab-developed digital PCR assay demonstrated excellent homogeneity (*F* = 0.54 < *F* crit = 2.39) and showed no significant changes in stability after storage at 4 or 25°C for up to 3 days or through three freeze-thaw cycles (one-sample *t*-test, *P* > 0.05 vs. −20°C control; see Table S4).

Similarly, the reference panel evaluated using two commercial CHIKV nucleic acid detection kits (fluorescence-based PCR method) also met all acceptance criteria. The CVs for positive samples (P1–P6) were all below 5% in both homogeneity and stability tests (see Table S5). Stability studies under identical conditions (4°C, 25°C, and freeze-thaw stress) confirmed that the panel’s performance was unaffected compared to the −80°C control (one-way analysis of variance, *P* > 0.05).

### Determination of the national standard assigned value

**Fig 1:**
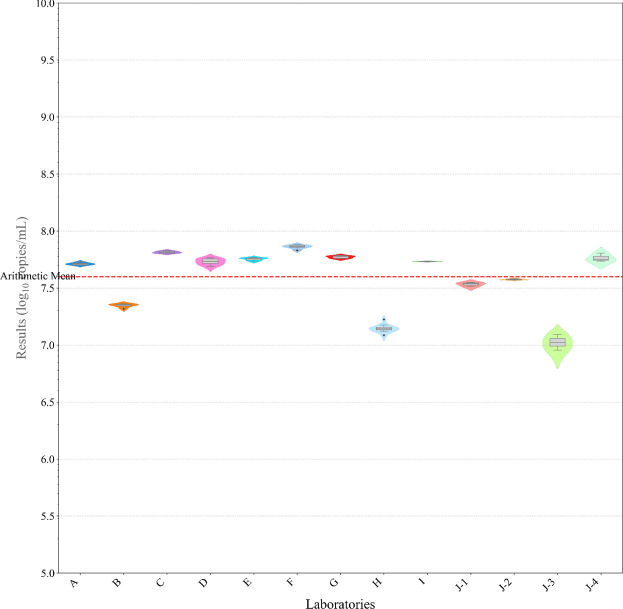
Multi-Platform dPCR value assignment results for the CHIKV national standard. Each data point represents an independent concentration value (log_10_ copies/mL) obtained from a specific dPCR platform used by a participating laboratory. For most laboratories, one data point is shown. Laboratory J employed four different dPCR platforms, contributing four independent data points as indicated. All values were derived from multiple replicate measurements performed on the reconstituted standard using the respective optimized in-house dPCR assays. The red dashed line indicates the arithmetic mean of all independent results (7.60 log_10_ copies/mL), which was established as the assigned value for the standard. The box represents the interquartile range (IQR, 25th to 75th percentiles); the horizontal line inside the box denotes the median; and the whiskers extend to 1.5 times the IQR.

The value assignment results from all participating laboratories are presented in Fig. 1. To obtain a robust estimate, raw concentration values were log₁₀-transformed, and the arithmetic mean of the transformed values was selected as the assigned concentration, consistent with the practice of major reference material producers (17, 18). The final assigned value of the candidate reference material is (7.60 ± 0.14) log₁₀ copies/mL, where the quoted uncertainty represents the expanded uncertainty (*k* = 2, 95% confidence interval [CI]).

### Performance characterization of the reference material system

Comprehensive evaluation using nine commercial CHIKV detection kits demonstrated excellent reactivity profiles. All kits achieved 100% positive agreement (detection of P1–P6), and all negative results were concordant with expectations. Repeatability testing of the national standard across two concentration levels demonstrated high precision, with all CV values below 5% (detailed data in Table 2). The LoD determined by serial dilution was no higher than the kits' claimed LoD, with a positive detection rate of at least 95% (Table 2).

**TABLE 2:** Summary of performance characterization results for the reference material system

Kit	Positive agreement (+/+)	Negative agreement (−/−)	Repeatability		LoD	
			Medium concentration	Low concentration	Experimentally determined LoD^*b*^	Claimed LoD
1	6/6	16/16	0.81%	1.73%	198.19	300
2	6/6	10/16	/^*a*^	1.58%	99.09	100
3	6/6	16/16	1.74%	1.63%	66.06	100
4	6/6	16/16	0.37%	1.75%	132.13	200
5	6/6	16/16	1.61%	2.31%	440.42	500
6	6/6	16/16	0.47%	0.48%	33.03	50
7	6/6	11/16	0.73%	0.97%	33.03	100
8	6/6	16/16	0.60%	1.79%	44.04	200
9	6/6	16/16	/	1.82%	132.13	200

^*a*^ “/” indicates that due to insufficient kit quantities, the corresponding tests were not conducted.

^*b*^ The concentration is measured in units of copies/mL; kits 2 and 7 are both multiplex nucleic acid testing for vector-borne viruses; and kits 2 and 9 are POCT products.

Based on the collaborative calibration results and relevant industry standards, the following quality control criteria were established for the CHIKV nucleic acid detection reference materials:

Positive agreement: All positive reference materials (P1–P6) must test positive for CHIKV nucleic acid, resulting in a positive agreement rate of 6/6.

Negative agreement: For all negative samples (N1–N16) within the kit’s detection scope, the results must be consistent with their expected pathogen types.

LoD: The LoD performance must meet the manufacturer’s claimed specifications.

Repeatability: For both medium-high and low-concentration samples, all detection results must be positive for CHIKV RNA, with a CV ≤5.0%.

## DISCUSSION

This study successfully developed a national reference material system for CHIKV nucleic acid testing comprising a quantified standard and a validation panel. A critical feature of these materials was their preparation from intact, inactivated viral particles rather than *in vitro* transcribed RNA or pseudovirus particles, thereby more accurately simulating the entire clinical testing process—from extraction to amplification. Notably, no international standard for CHIKV nucleic acid testing has been established by the WHO to date, although related development efforts may be underway. Consequently, the reference material system developed in this study not only fills a critical gap in domestic quality assurance but also provides a valuable resource and technical reference (e.g., in terms of value assignment) for future international standardization efforts.

Comprehensive evaluation data confirmed excellent homogeneity and stability of the reference material system. The assigned values determined through a multi-laboratory collaborative study using digital PCR ensure metrological traceability and accuracy. Furthermore, the reference panel incorporates strains from both the Asian and ECSA genotypes, thereby covering major circulating genetic variants, which effectively mitigates the risk of false-negative results due to genetic diversity. Performance validation across nine commercial kits demonstrated 100% positive agreement, a detection rate ≥95% at the claimed LoD, and a repeatability CV of less than 5%. These results collectively confirm the robust analytical performance and practical utility. Although all nine commercial kits achieved the ≥95% positivity criterion at their claimed LoD, minor variations in the exact positivity rate and the determined LoD concentration were observed. These subtle differences likely stem from several assay-specific factors, including: (i) the genomic region targeted by primers and probes (e.g., E1, NSP1, and NSP2), (ii) amplification efficiency and reaction mix optimization, (iii) the efficiency of viral RNA extraction from the lyophilized matrix, and (iv) potential differences in the inclusivity of primer/probe sequences across genotypes. The establishment of this common reference material system provides a standardized platform to systematically investigate and optimize these variables, ultimately contributing to improved assay harmonization.

Several limitations should be noted. First, the current panel does not include the West African genotype; future iterations should incorporate a broader range of lineages to enhance global applicability. Second, lyophilization has not been fully implemented for the entire reference material system—optimization of preparation techniques is planned to improve long-term stability. Third, the LoD evaluation did not fully adhere to the CLSI EP17-A2 guidelines; subsequent studies will incorporate more standardized protocols to address this gap. Fourth, this study focused only on qualitative validation of CHIKV nucleic acid detection assays and did not perform systematic evaluation of quantitative performance across commercial kits using the national standard as a calibrator. Linear regression is the appropriate statistical method for quantifying platform linearity, accuracy, and inter-assay comparability. Further quantitative characterization to establish metrological traceability of the national standard as a primary quantitative reference material will be a key direction for future investigation. Implementation of this national reference material system is expected to optimize full-lifecycle quality management of CHIKV nucleic acid detection kits in China. Major applications include: (i) serving as a mandatory reference standard for the National Medical Products Administration (NMPA) in registration evaluation and technical review to verify analytical sensitivity, specificity, and precision; (ii) acting as a core reference for inter-laboratory comparisons to enhance the comparability and reliability of detection results; and (iii) providing a metrologically traceable basis for value assignment by manufacturers during product development, post-market verification, and manufacturing process validation.

Notwithstanding these limitations, the reference material system established in this work represents a significant enhancement of the quality control capacity for CHIKV nucleic acid detection in China. They provide a scientifically robust measurement standard for the development, evaluation, and validation of related kits and lay a solid foundation for future updates of the reference materials and contribute to the broader goal of global standardization.

## SUPPLEMENTAL MATERIAL

Supplemental tables jcm.01543-25-s0001.docx Tables S1 to S5.

ASM does not own the copyrights to Supplemental Material that may be linked to, or accessed through, an article. The authors have granted ASM a non-exclusive, world-wide license to publish the Supplemental Material files. Please contact the corresponding author directly for reuse.
