# Supplementary material for: Development and validation of a national reference material system for quality control of chikungunya virus nucleic acid detection assays
Source: J Clin Microbiol. 2026 May 6;64(6):e01543-25. doi: 10.1128/jcm.01543-25 (PMC13251405; doi:10.1128/jcm.01543-25)
Supplement: Supplemental tables — Tables S1 to S5. [file jcm.01543-25-s0001.docx]

**Supplementary Table 1. Critical Process Parameters for Lyophilization of the CHIKV National Standard**

| **Process Stage** | **Parameter** | **Value/Description** |
| --- | --- | --- |
| Pre-freezing | Final temperature | -45℃ |
|  | Holding time | 2 h |
| Primary Drying | Shelf temperature | -25℃ |
|  | Vacuum level | 0.1 mbar |
|  | Holding time | 14 h |
| Secondary Drying | Shelf final temperature | 35℃ |
|  | Vacuum level | 0.01 mbar |
|  | Holding time | 3.5 h |
| Post-lyophilization | Back-filling gas | Dry nitrogen |
|  | Filling pressure | 0.2 pa |
|  | Sealing method | Crimp sealing |

All parameters were strictly controlled to ensure the stability, homogeneity and reproducibility of the lyophilized CHIKV national standard.

**Supplementary Table 2. Participating Laboratories and Digital PCR Methodologies for Value Assignment**

| **Lab** | **Brand** | **Methodology** | **Target Gene** | **Coverage** |
| --- | --- | --- | --- | --- |
| A | Qiagen | Chip-based | NSP1 | 899~1320 |
| B | Stilla Nacia | Droplet-based | E1 | 10100~10300 |
| C | Bio Rad | Droplet-based | NSP1 | 280~1380 |
| D | Bio Rad | Droplet-based | E1 | 10788~11036 |
| E | Targeting One | Droplet-based | NSP4 | 6210~6350 |
| F | Qiagen | Chip-based | NSP2 | 2601~2675 |
| G | Maccura | Droplet-based | E1 | 10381~10801 |
| H | Forevergen | Droplet-based | E1 | 10237~10620 |
| I | Targeting One | Droplet-based | NSP1 | 4400~4700 |
| J | Maccura | Droplet-based | E1 | 10296~10500 |
|  | Targeting One | Droplet-based |  |  |
|  | Bio Rad | Droplet-based |  |  |
|  | Thermo | Chip-based |  |  |

Coverage refers to the nucleotide positions on the CHIKV reference genome (JX088705.1) covered by the primers/probes used in the dPCR assay.

Li XF, Jiang T, Deng YQ, Zhao H, Yu XD, Ye Q, Wang HJ, Zhu SY, Zhang FC, Qin ED, Qin CF. 2012.Data from “Complete genome sequence of a chikungunya virus isolated in Guangdong, China.” GenBank https://www.ncbi.nlm.nih.gov/nucleotide/JX088705 (accession no. JX088705). {Accession number.}

**Supplementary Table 3. Details of Commercial CHIKV Nucleic Acid Detection Kits Used for Collaborative Calibration of the National Reference Material System**

| **Manufacturer** | **Detection kit** |
| --- | --- |
| Beijing Wantai Biological Pharmacy Enterprise Co., Ltd. | CHIKV/ZIKA/DENV Nucleic Acid Detection Kit (Fluorescence PCR Method) |
| Guangzhou Wondfo Biotech Co., Ltd. | Chikungunya Virus Nucleic Acid Detection Kit (Fluorescence PCR Method) |
| Beijing Wantai Biological Pharmacy Enterprise Co., Ltd. | Chikungunya Virus Nucleic Acid Detection Kit (Fluorescence PCR Method) |
| Jiangsu Shuoshi Biological Technology Co., Ltd. | Chikungunya Virus Nucleic Acid Detection Kit (Fluorescence PCR Method) |
| *Beijing BOE Zhiwei Microbiology Technology Co., Ltd.* | *Multiplex Nucleic Acid Detection Kit for CHIKV/ZIKA/DENV/YFV (Fluorescence PCR Method)* |
| Guangdong Hexin Health Technology Co., Ltd. | Chikungunya Virus Nucleic Acid Detection Kit (Fluorescence PCR Method) |
| Beijing BGI-GBI Biotech Co., Ltd. | Chikungunya Virus Nucleic Acid Detection Kit (Fluorescence PCR Method) |
| Shanghai Bojie Medical Technology Co., Ltd. | Chikungunya Virus Nucleic Acid Detection Kit (Fluorescence PCR Method) |
| *Guangzhou Wondfo Biotech Co., Ltd.* | *Chikungunya Virus Nucleic Acid Detection Kit (Fluorescence PCR Method)* |

All collaborative calibration reagents used in this study are for research use only and have not been approved by the National Medical Products Administration (NMPA) of China. Due to confidentiality agreements with the participating manufacturers, target gene information for each manufacturer has been removed, and the collaborative calibration results presented in Table 2 cannot be linked to specific manufacturers. No one-to-one correspondence between the listed manufacturers and the calibration results is provided. Items shown in italics are POCT products. The list of manufacturers is available from the corresponding author upon reasonable request.

**Supplementary Table 4. Short-term stability evaluation results for national standard**

| **Conditions** | **-20℃** | **4 ℃ 1 Day** | **4 ℃ 2 Days** | **4 ℃ 3 Days** | **RT^1^ 1 Day** | **RT 2 Days** | **RT 3 Days** | **FT^2^ 1 Time** | **FT 2 Times** | **FT 3 Times** |
| --- | --- | --- | --- | --- | --- | --- | --- | --- | --- | --- |
| TEST 1 | 3.26E+07 | 3.00E+07 | 3.20E+07 | 3.42E+07 | 2.69E+07 | 2.91E+07 | 3.26E+07 | 3.29E+07 | 4.21E+07 | 2.92E+07 |
| TEST 2 | 3.15E+07 | 2.99E+07 | 3.46E+07 | 3.76E+07 | 2.86E+07 | 3.00E+07 | 3.13E+07 | 3.63E+07 | 3.56E+07 | 3.11E+07 |
| TEST 3 | 3.12E+07 | 3.28E+07 | 3.24E+07 | 3.40E+07 | 3.14E+07 | 3.37E+07 | 2.90E+07 | 3.17E+07 | 3.16E+07 | 3.22E+07 |
| Average | 3.18E+07 | 3.09E+07 | 3.30E+07 | 3.53E+07 | 2.90E+07 | 3.09E+07 | 3.10E+07 | 3.36E+07 | 3.64E+07 | 3.08E+07 |
| *P* | N/A | 0.23 | 0.08 | 0.09 | 0.06 | 0.18 | 0.49 | 0.13 | 0.23 | 0.08 |

^1^Room temperature; ^2^Freezing and thawing; copies/mL measured on the Bio-Rad QX200 system.

**Supplementary Table 5. Statistical analysis results on the homogeneity and stability of the National Reference Panel**

| **ID** | **CV(%)^1^** | **CV(%)^2^** | **CV(%)^3^** | **CV(%)^4^** |
| --- | --- | --- | --- | --- |
| P1 | 1.34% | 0.83% | 1.11% | 1.44% |
| P2 | 0.89% | 0.47% | 0.88% | 1.81% |
| P3 | 2.65% | 1.76% | 0.72% | 2.95% |
| P4 | 1.52% | 1.14% | 1.16% | 2.82% |
| P5 | 1.81% | 1.34% | 1.76% | 3.28% |
| P6 | 0.95% | 1.88% | 1.47% | 3.25% |
| N1~N16 | N/A | N/A | N/A | N/A |

The four columns of CV (%) values correspond to: 1. material homogeneity assessment; 2. short-term stability testing after storage at 4 ℃ for 3 days; 3. short-term stability testing after storage at room temperature for 3 days; 4. short-term stability testing after 3 freeze-thaw cycles; N1~N16 represent negative reference material, which contain no target CHIKV nucleic acid; thus, CV values cannot be calculated (marked as N/A).
